# No Vegetative and Fecundity Fitness Cost Associated with Acetyl-Coenzyme A Carboxylase Non-target-site Resistance in a Black-Grass (*Alopecurus myosuroides* Huds) Population

**DOI:** 10.3389/fpls.2017.02011

**Published:** 2017-11-28

**Authors:** Eshagh Keshtkar, Solvejg K. Mathiassen, Per Kudsk

**Affiliations:** ^1^Department of Agroecology, Faculty of Science and Technology, Aarhus University, Slagelse, Denmark; ^2^Department of Agronomy, Faculty of Agriculture, Tarbiat Modares University, Tehran, Iran

**Keywords:** ACCase, biomass, black-grass, fitness penalty, NTSR, potential seed production

## Abstract

Attention should be devoted to weeds evolving herbicide resistance with non-target-site resistance (NTSR) mechanism due to their unpredictable resistance patterns. Quantification of fitness cost can be used in NTSR management strategies to determine the long-term fate of resistant plants in weed populations. To our knowledge, this is the first report evaluating potential fecundity and vegetative fitness of a NTSR black-grass (*Alopecurus myosuroides* Huds), the most important herbicide resistant weed in Europe, with controlled genetic background. The susceptible (S) and NTSR sub-populations were identified and isolated from a fenoxaprop-*P*-ethyl resistant population by a plant cloning technique. Using a target-neighborhood design, competitive responses of S and NTSR black-grass sub-populations to increasing density of winter wheat were quantified for 2 years in greenhouse and 1 year in field. Fitness traits including potential seed production, vegetative biomass and tiller number of both sub-populations significantly decreased with increasing density of winter wheat. More importantly, no statistically significant differences were found in fitness traits between S and NTSR sub-populations either grown alone (no competition) or in competition with winter wheat. According to the results, the NTSR black-grass is probably to persist in field even in the cessation of fenoxaprop-*P*-ethyl. So, effective herbicide resistant management strategies are strongly suggested to prevent and stop the spread of the NTSR black-grass, otherwise NTSR loci conferring resistance to a range of herbicides in black-grass will persist in the gene pool even in the absence of herbicide application. Consequently, herbicide as an effective tool for control of black-grass will gradually be lost in fields infested by NTSR black-grass.

## Introduction

Herbicides are the most reliable, applicable and cost-effective method of weed control (Moss, 2010; Keshtkar, 2015; Owen, 2016). However, the continuous use of herbicides for many years have led to widespread problems with herbicide resistance. Resistance to herbicides is a major threat to the sustainability of agricultural industry with now 251 herbicide resistance weed species being reported worldwide (Heap, 2017).

Black-grass (*Alopecurus myosuroides* Huds), a common winter-annual grass weed species, can reduce crops yield substantially (Lutman et al., 2013). Black-grass has been reported in 37 countries and it is the most important weed species in European cereals especially it is a major weed in the United Kingdom, France, and Germany (Holm et al., 1997; Chauvel et al., 2002). It is also the most important herbicide resistant weed species in Europe (Moss et al., 2007; Lutman et al., 2013). Black-grass has evolved resistance to seven different sites of action including group A (1), B (2), C1 (5), C2 (7), K1 (3), K3 (15), and N (8) (Heap, 2017) and both target-site resistance (TSR) and non-target-site resistance (NTSR) mechanisms were detected among black-grass populations (Délye, 2005). TSR mechanism to group A herbicides (ACCase inhibitors) were reported in black-grass ACCase gene at five codons including Ile-1781, Trp-2027, Ile-2041, Asp-2078 and Gly-2096 (Kaundun, 2014). Target-site mutation conferring resistance to group B herbicides (ALS inhibitors) were observed in black-grass ALS gene at position Pro-197 and Trp-574 (Hull et al., 2008; Tranel et al., 2017). As one of several mechanisms endowing NTSR, enhanced herbicide metabolism mediated by cytochrome P 450 monooxygenases (P-450) and glutathione *S*-transferase (GST) was reported in black-grass populations evolving resistance to different group of herbicides (Hall et al., 1995; Cummins et al., 1999; Preston, 2004; Délye, 2005). It was also confirmed that higher activity of *O*-glucosyltransferases (OGTs) endows NTSR (Brazier et al., 2002). As our literature review revealed, other NTSR mechanisms such as reduced penetration and translocation have not been reported in black-grass (Hall et al., 1995; Menendez and De Prado, 1996), meaning that enhanced metabolism is the main mechanism endowing NTSR in black-grass.

Herbicide resistance strategies should aim to prevent or delay the evolution of herbicide resistance and whenever resistant weed species appear, management strategies should aim to reduce the number and frequency of resistant plants within populations. According with general eco-evolutionary theories resistant (R) plants are expected to be less fit than susceptible plants in the absence of selection pressure (no herbicide) (Vila-Aiub et al., 2009b; Vila-Aiub et al., 2011). This hypothesis holds true for weed biotypes carrying the *Gly264 psbA* gene endowing resistance to triazine herbicides (Gronwald, 1994). It can, however, not be generalized to other cases of resistance. In some cases no resistance costs were reported as for glyphosate-resistant palmer amaranth (*Amaranthus palmeri*) (Giacomini et al., 2014; Vila-Aiub et al., 2014), while in other cases, surprisingly, a higher fitness was reported, e.g., for a sethoxydim-resistant green foxtail (*Setaria italic*) (Wang et al., 2010). Thus, it is necessary to evaluate fitness costs of each case of resistance individually (Lehnhoff et al., 2013) to develop robust resistance management programs (Park et al., 2004; Vila-Aiub et al., 2011). For instance, Gly-2078 ACCase allele conferring resistance to fenoxaprop-*P*-ethyl and clodinafop-propargyl in a black-grass population was associated with a fitness cost, hence using competitive crops like alfalfa in crop rotation programs can strongly hamper the development and seed production of plants carrying the R allele compared with wild type plants (Menchari et al., 2008).

It has been stressed by several researchers that factors like the genetic background, environmental condition, and resource competition should be considered in fitness studies (Holt, 1990; Menchari et al., 2008; Vila-Aiub et al., 2009b). Among these, control of genetic background of S and R biotypes is one of the main obstacles assessing fitness cost. Vila-Aiub et al. (2009b) found that the genetic background was only controlled in 25% of the published fitness studies. In many studies S and R populations originating from different geographical locations with different genetic background were used and results from such studies might be inconclusive as different populations may differ genetically at a number of loci other than resistant locus or loci (Keshtkar et al., 2017).

Fitness can be defined as reproductive success of a plant in a given environment (Holt, 1990), i.e., the number of offspring sent to next generations by an individual compared to another (Vila-Aiub et al., 2009b). Therefore, seed production is a crucial parameter determining fitness (Menchari et al., 2008; Vila-Aiub et al., 2009b). The seed production of black-grass correlated to the number of tillers (Maréchal et al., 2012). Around 50% of produced caryopsis (i.e., seeds) can be viable (Pye, 2000) and viability of seeds is affected by the time of seed shedding (Moss, 1983). In a life cycle model of black-grass, it was also assumed that viability of the produced seeds are 55%, while half of the viable seeds are lost due to predation, seed decay in soil and germination before cultivation (Cavan et al., 2000). Cropping systems can also affect seed production and density of black-grass (Chauvel et al., 2001), especially spring crops such as spring wheat reducing up to 88% of black-grass population (Lutman et al., 2013).

In contrast to many studies that only evaluated vegetative output of plants in non-competition condition, in this study we monitored the whole life cycle of the R and S plants (i.e., both vegetative and reproductive potential) under both competitive and non-competitive conditions. Studies on resistance costs of NTSR are sparse (Vila-Aiub et al., 2005a). In a previous study, we evaluated the costs of NTSR in a black-grass population on germinability and seedling emergence under contrasting temperature regimes (optimal and low) and different sowing depths. In comparison to the S sub-population, around 24 h delay was observed in seedling emergence of the R sub-population, where the seeds were grown at suboptimal conditions. Importantly, the seedling emergence of the R sub-population was around four-fold lower than the corresponding S sub-population. These results suggested that manipulation of agronomy practices such as delayed drilling of winter wheat and avoiding no-tillage can cause unfavorable conditions for the R sub-population (Keshtkar et al., 2017). In that and the present study, we followed the experimental protocol proposed by Vila-Aiub et al. (2011) previously described by Vila-Aiub et al. (2005b) and Pedersen et al. (2007) to control genetic background of plant material through selection of R and S phenotypes within a single population. The objective of present study was to assess the vegetative and reproductive capacity under non-competitive and competitive condition of the same NTSR black-grass sub-population. The response of the S and NTSR sub-populations were evaluated in a target-neighborhood design at increasing densities of winter wheat in greenhouse and field conditions. To our knowledge, this is the first study comparing vegetative and reproductive output of S and NTSR black-grass sub-populations sharing random homogeneous genetic background.

## Materials and Methods

### Plant Material

A known fenoxaprop-*P*-ethyl NTSR black-grass population (population ID914) originating from a field near Odense on the island of Funen in Denmark (54° 57′ 21.93″ N 10° 36′ 39.53″ E) was used in this study. Enhanced herbicide metabolism assumed to be the mechanism endowing NTSR in the studied population ID914, as it is common in black-grass populations (Hall et al., 1995; Cummins et al., 1999; Preston, 2004; Délye, 2005) and was also expected in 75% of ACCase (i.e., fenoxaprop-*P*-ethyl and clodinafop-propargyl)-resistant black-grass populations in France (Délye et al., 2007). Recently, it was also demonstrated that enhanced metabolism plays an important role in developing resistant black-grass populations (Kaiser and Gerhards, 2015). Also, other NTSR mechanisms including reduced penetration and translocation have not been reported in black-grass (Hall et al., 1995; Menendez and De Prado, 1996). The population ID914 was highly resistant to fenoxaprop-*P*-ethyl [resistance index (RI) > 64], moderately resistant to pendimethalin and flupyrsulfuron-methyl-sodium (RI > 10), and slightly resistant to prosulfocarb (RI > 2.5) (Keshtkar et al., 2015).

To select susceptible and NTSR phenotypes within the population ID914 a plant cloning technique, the so-called “Single Population Protocol” described by Vila-Aiub et al. (2005b) and Pedersen et al. (2007), was used as in a previous study (Keshtkar et al., 2017). Briefly, the seedlings of populations ID914 at the 2–3 tillering stage were propagated asexually by a dividing method. The parent plants and their corresponding clones were numbered for identification. The clones were sprayed with fenoxaprop-*P*-ethyl at the 3–4 leaf stage and classified as S (dead plants), partly resistant (PR, alive but no regrowth) and R (alive and vigorously regrowth). The corresponding parent plants were then identified according to their number. The PR parent plants were discarded, while the S and R parent plants were grown in separate tables in a cold greenhouse for vernalization. The plants surrounded by a polyethylene pollen-proof enclosure to prevent cross-pollination between the S and NTSR parent plants until seed production in mid-July 2012. Seeds were harvested and to break primary seed dormancy they were placed at high temperature (35 ± 2°C) for 6 weeks as suggested by Moss (1999) and then stored at a constant temperature of 4°C until the onset of the experiments. To confirm resistance status of the R and S sub-populations selected within the mother population (i.e., population ID914), a dose-response experiment was carried out using different dose of fenoxaprop-*P*-ethyl. Results of the dose-response experiment confirmed that the plant cloning technique selected the R and S sub-populations very well as the R sub-population was highly resistant to fenoxaprop-*P*-ethyl, while the S one was sensitive (Keshtkar et al., 2017). Molecular assays showed that none of the known mutations conferring target site resistance to ALS and ACCase inhibiting herbicides were present in either the original parent population, i.e., the population ID914 (Keshtkar et al., 2015) or in the S and R sub-populations, suggesting that the mechanism of resistance was NTSR (Keshtkar et al., 2017). Also, herbicides with four different sites of action failed to control the parent population ID914 (Keshtkar et al., 2015), providing more evidence that the mechanism of resistance was NTSR (Keshtkar et al., 2017). As it was described above, it is expected that NTSR mechanism is due to enhanced herbicide metabolism.

### Greenhouse Experiment

A target-neighborhood design was used to compare the vegetative and reproductive ability of the S and R sub-populations (target plant), in response to increasing densities of winter wheat (neighbor plant) where winter wheat was either at the 2-leaf or the 3–4 leaf stage.

**FIGURE 1:**
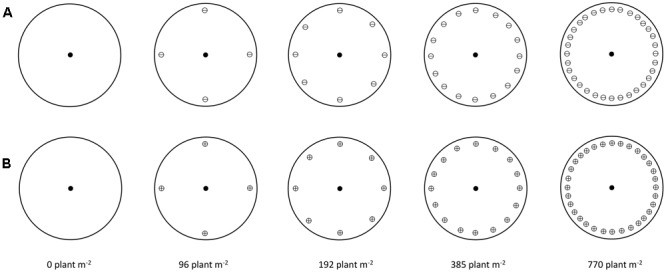
Schematic illustration of target-neighborhood experiment design used to evaluate the competitive responses of S and NTSR sub-populations to increasing density of winter wheat at **(A)** 2-leaf growth stage of winter wheat (**⊖**) and **(B)** 3-4 leaf growth stage of winter wheat (⊕). Symbols in the middle of circles represent black-grass plants (target plants) at 2-leaf growth stage (●). Symbols close to edge of circle represent winter wheat plants (neighbor plants).

Five winter wheat (cv. Herford) densities of 0, 96, 192, 385, and 770 plants m^-2^ were established by sowing 0, 4, 8, 16, and 32 plants in pots (23 cm diameter × 30 cm height). The pots were filled with a potting mixture consisting of soil, peat, and sand (2:1:1 w/w/w) containing all necessary micro and macro nutrients. A template was used to achieve the same distance between each neighbor plant and the target plant and a uniform distance among neighbor plants (**Figure 1**). Additional winter wheat plants were grown in plastic boxes and transplanted at the 2-leaf growth stage to the pots where winter wheat seeds failed to germinate or germinated late.

Uniform sized non-dormant seeds of the R and S black-grass sub-populations were pre-germinated in 9-cm Petri-dishes containing three cellulose filter papers (Whatman No. 1) covered by one glass-fiber filter paper (Whatman GF/A). Seven ml of distilled water was added to each Petri-dish and the dishes were placed at 17/10°C day/night with 14/10 h light/darkness photoperiods with a Photosynthetic Photon Flux Density of 175 μmol m^-2^ s^-1^. The seedlings with approximately 1 cm length were transplanted into small Jiffy pots and grown till they reached the 2-leaf stage. Uniform 2-leaf stage black-grass seedlings were re-transplanted into the center of each pot when winter wheat was either at the 2-leaf (**Figure 1A**) or the 3–4-leaf growth stage (**Figure 1B**). The 3–4-leaf growth stage treatment was included in the study as we wanted to introduce an additional stress on black-grass by exposing the plants to competition from the more competitive wheat plants, i.e., the 3–4 leaf growth. The 2-leaf growth stages of both wheat and black-grass mimics the scenario where black-grass seeds germinate at the same time as winter wheat, while the 3–4 leaf growth stage of winter wheat reflects a scenario where black-grass seeds germinate later than winter wheat which is not uncommon in black-grass germinates between September and December pots were sub-irrigated to maintain field capacity and a liquid fertilizer was applied as required. The experiment was initiated in the late December 2012 and the pots were kept in a cold greenhouse until spring to vernalize plants and it was terminated in June 2013. The experiment was repeated in the growing season 2013/14.

Above-ground biomass of target and neighbor plants was determined at the tillering stage of black-grass (first harvest time) and the stem elongation stage (second harvest time) by cutting plants at the soil surface. The plants were oven dried for 24 h at 80°C and dry weight was recorded. In addition, the number of tillers of black*-*grass was measured. Three replicate pots per treatment were harvested at each harvest time.

Three pots per treatment were kept in the greenhouse for evaluation of reproductive ability. Seeds of black-grass mature and shed over a long period, hence it was not possible to measure seed production. Instead, the potential seed production of plants was estimated indirectly, as suggested by Melander (1995). The heads from each pot were cut immediately after emergence (before flowering), kept in small mesh plastic bags at greenhouse temperature to be dried and finally, the cumulative weight of heads was measured. Around 80 heads were used to calculate linear relationship between seeds per head and head weight. The estimated *R*^2^ values for the 2012/13 and 2013/14 experiments were 0.81 and 0.73, respectively. Above-ground biomass was measured at the end of the experiment (third harvest time). Aphids and powdery mildew were controlled twice with the insecticide (imidacloprid (Confidor WG 70; Bayer)) and the fungicide (metrafenon (Flexity; BASF)).

### Field Experiment

A target-neighborhood experiment was also conducted in a field in 2013/14 almost similar to the greenhouse experiments. A field (50.5 × 17.5 m) was sown with winter wheat (cv. Herford) on 6 September 2013. Eighty small plots (50 cm × 50 cm) were left bare within the field. The distance between plots was at least 2.5 m. Similar to the greenhouse experiments, five winter wheat densities (0, 96, 192, 385, and 770 plants m^-1^) were established by sowing 0, 4, 8, 16, and 32 winter wheat in the center of each plot on 11 September 2013 using the same templates as for the greenhouse trial. The S and NTSR black-grass seedlings were grown outdoors using the same method as for the glasshouse experiments. At the 3–4-leaf stage of winter wheat, uniform 3–4-leaf stage seedlings of the two sub-populations of black-grass were transplanted into the center of each plot.

Additional plants of both black-grass sub-populations and winter wheat were grown in a nursery in the same field. Poorly established plants were replaced by new seedlings supplied from the nursery either end of October 2013 and or end of March 2014. The number of replaced seedlings were the same for NTSR and S sub-populations. All plots were sprayed with imidacloprid (Confidor WG 70; Bayer) in late October 2013 to prevent damage from wireworm (*Agriotes* spp.). To avoid pollen dispersal from the NTSR plants to the natural stand of black-grass in the surrounding fields the experiment was terminated at the stem-elongation stage of black-grass (before heading stage) by cutting the plants at the soil surface on 16 May 2014. Dry matter and number of tillers were recorded at the mentioned growth stage.

### Statistical Analysis

The greenhouse trials were designed as split-split factorial experiments with four factors (i.e., two black-grass sub-populations × five winter wheat densities × three harvesting times × two growth stages of winter wheat), where winter wheat growth stage was considered as main plot and winter wheat density as subplot. A Completely Randomized Design (CRD) was used with three replicates per treatment, resulting in 180 pots per experiment.

The field trial was a factorial experiment with two factors (two black-grass sub-populations × five winter wheat densities) arranged as a Randomized Complete Block Design (RCBD). There were four replicates per treatment giving 40 plots in total.

Regression analysis was carried out to evaluate the competitive responses of the S and NTSR sub-populations to increasing density of winter wheat. Specifically, a non-linear hyperbola model was fitted to data, the model equation used was:

$$\begin{array}{c} Y=\frac{a}{1+\left[\frac{x}{ED_{50}}\right]} \end{array}$$

where *Y* denotes the black-grass end-point (biomass, tiller number, and potential seed production), *x* represents density of winter wheat (plant m^-2^), *a* is the upper limit or mean response when winter wheat density is zero (no crop competition), the parameter *ED_50_* is the effective density of wheat reducing black-grass end-point by 50%. The sub-populations were compared in terms of the parameters *a, ED_50_*, and *ED_90_* (the effective density of wheat reducing black-grass end-point by 90%) by means of *post hoc t*-tests. For each harvest time, the model was fitted simultaneously to the data of both sub-populations. Wherever the assumption of homogeneity of variance was not met a transform-both-sides approach (Box–Cox data transformation) was applied (Carroll and Ruppert, 1988; Ritz et al., 2016). The R statistical software with the add on package *drc* was used for the statistical analysis and making graphs (Ritz and Streibig, 2005; R Core Team, 2013).

## Results

### Greenhouse Experiments

Data were analyzed separately for each year as significant differences were found in the estimated parameters between years. The results showed that the biomass, tiller number, and potential seed production of both the R and S sub-populations significantly decreased with increasing density of winter wheat (**Tables 1, 2** and Supplementary Figures S1–S3). This response was observed at both growth stages of winter wheat in both years. As expected, suppression of black-grass by increasing densities of winter wheat was more pronounced at the 3–4 leaf stage of winter wheat, i.e., when the winter wheat plants were more developed and, thus, more competitive than the black-grass plants.

**Table 1:** Competitive response of S and NTSR sub-populations selected within a black-grass population (ID914), to increasing density of winter wheat at two different growth stages of winter wheat (GS-I; 2-leaf stage, GS-II; 3–4 leaf stage) in the 2012/13 greenhouse experiment.

Plant traits	Harvest time	*ED_50_* (winter wheat plants m^-2^)^a^						*ED_90_* (winter wheat plants m^-2^)^b^						*D*^c^					
		GS-I			GS-II			GS-I			GS-II			GS-I			GS-II		
		*S*	*R*	*S*	*R*	*S*	*R*	*S*	*R*	*S*	*R*	*S*	*R*
Biomass	H 1	270 (70.7)	*ns*	222 (51.8)	44 (9.8)	*ns*	56 (11.9)	2427 (635.9)	*ns*	1998 (466.6)	393 (88.1)	*ns*	505 (107.5)	1.0 (0.11)	*ns*	1.1 (0.12)	0.8 (0.11)	*ns*	0.9 (0.12)
	H 2	74 (22.2)	*ns*	79 (23.3)	15 (4.0)	*ns*	16 (4.1)	667 (200.0)	*ns*	713 (209.3)	136 (36.0)	*ns*	144 (37.2)	9.0 (1.71)	*ns*	10.4 (1.91)	8.0 (1.58)	*ns*	7.8 (1.51)
	H 3	46 (12.1)	*ns*	76 (22.4)	11 (2.9)	*ns*	11 (2.8)	417 (109.1)	*ns*	682 (201.6)	101 (26.3)	*ns*	100.9 (25.3)	146.2 (24.60)	*ns*	140.2 (24.41)	131.2 (22.80)	*ns*	149.9 (25.19)
Tiller No.	H 1	281 (65.0)	*ns*	347 (85.9)	67 (14.3)	*ns*	97 (20.9)	2529 (585)	*ns*	3128 (773)	603 (129)	*ns*	873 (189)	19.5 (1.90)	*ns*	18.9 (1.82)	14.7 (1.83)	*ns*	13.7 (1.66)
	H 2	71 (13.8)	*ns*	92 (17.2)	28 (4.9)	*ns*	26 (4.4)	637 (123.80	*ns*	826 (154.70	251 (44.3)	*ns*	235 (39.7)	56.6 (6.94)	*ns*	67.0 (7.63)	40.8 (5.17)	*ns*	44.1 (5.42)
Potential seed production	H 3	42 (8.6)	*ns*	58 (14.1)	11 (2.6)	*ns*	14 (3.4)	376 (77.4)	*ns*	519 (126.8)	102 (23.3)	*ns*	129 (30.6)	70061 (8429.9)	*ns*	56343 (7608.4)	67984 (8366.2)	*ns*	53136 (7071.2)

*The parameters obtained by fitting a nonlinear hyperbola model to the data of the 2012/13 greenhouse experiment. Values in parentheses represent standard error of the estimated parameters. ^a^Effective density of wheat reducing response of target plants by 50%. ^b^Effective density of wheat reducing response of target plants by 90%. ^c^Mean response of target plants in the absence of winter wheat. *^ns^*Non-significant (no significant differences between the R and S sub-populations in each growth stages of winter wheat).*

**Table 2:** Competitive responses of S and NTSR sub-populations selected within a black-grass population (ID914), to increasing density of winter wheat at two different growth stages of winter wheat (GS-I; 2-leaf stage, GS-II; 3-4 leaf stage) in the 2013/14 greenhouse experiment.

Plant traits	Harvest time	ED_50_ (winter wheat plants m^-2^)^a^						ED_90_ (winter wheat plants m^-2^)^b^						D^c^					
		GS-I			GS-II			{GS-I			GS-II			GS-I			GS-II		
		*S*	*R*	*S*	*R*	*S*	*R*	*S*	*R*	*S*	*R*	*S*	*R*
Biomass	H 1	118 (26.7)	*ns*	145 (35.6)	33 (6.5)	*ns*	25 (4.6)	1061 (240.4)	*ns*	1305 (320.2)	299 (58.7)	*ns*	226 (41.4)	0.75 (0.087)	*ns*	0.61 (0.0740	0.75 (0.088)	*ns*	0.96 (0.104)
	H 2	42 (25.5)	*ns*	52 (35.5)	5 (2.4)	*ns*	4 (1.9)	375 (229.1)	*ns*	469 (319.3)	41 (22.1)	*ns*	33 (17.7)	11 (5.3)	*ns*	9 (4.7)	18 (8.6)	*ns*	14 (6.9)
	H 3	42 (20.0)	*ns*	82 (40.5)	4 (2.1)	*ns*	5 (2.6)	375 (180.4)	*ns*	737 (364.8)	37 (18.5)	*ns*	49 (23.0)	67 (20.1)	*ns*	64 (19.0)	94 (27.6)	*ns*	103 (29.3)
Tiller No.	H 1	197 (67.8)	*ns*	230 (78.8)	37 (10.3)	*ns*	36.7 (10.2)	1769 (610.0)	*ns*	2068 (709.4)	332 (93.0)	*ns*	331 (92.3)	10 (1.9)	*ns*	10 (1.8)	18 (4.0)	*ns*	17 (3.8)
	H 2	49 (16.9)	*ns*	46 (17.0)	10 (3.0)	*ns*	14 (4.2)	442 (152.7)	*ns*	410 (153.6)	89.8 (27.3)	*ns*	125 (37.9)	63 (15.6)	*ns*	66 (17.5)	80 (19.7)	*ns*	70 (16.7)
Potential seed production	H 3	41 (30.1)	*ns*	68 (50.3)	3 (1.9)	*ns*	3 (1.8)	368 (271.2)	*ns*	608 (453.1)	26 (17.4)	*ns*	25 (16.2)	49123 (26776.5)	*ns*	47477 (24509.1)	59762 (33022.0)	*ns*	75589 (40936.7)

*The parameters obtained by fitting a nonlinear hyperbola model to the data of the 2013/14 greenhouse experiment. Values in parentheses represent standard error of the estimated parameters. ^a^Effective density of wheat reducing response of target plants by 50%. ^b^Effective density of wheat reducing response of target plants by 90%. ^c^Mean response of target plants in the absence of winter wheat. ^ns^Non-significant (no significant differences between the R and S sub-populations in each growth stages of winter wheat).*

More importantly, at the maturity stage (third harvest time) no statistically significant differences were found in fitness traits (potential seed production and final biomass) between the R and S sub-populations either grown alone (no competition) or in competition with winter wheat in either year. A similar result was observed at other growth stages including tillering and stem-elongation stages, i.e., there were no statistically significant differences between the R and S sub-populations. A similar trend to the *ED*_50_ values was observed for number of tillers, biomass and potential seed production and no significant differences were found between the corresponding *ED*_90_ values at none of the harvest times, winter wheat growth stages and either year (**Tables 1, 2**). Overall winter wheat density had more effect on biomass of black-grass at the last harvest time compared to the first harvest time reflecting an increased crop competition in the late growing season. As no significant differences were observed between the R and S sub-populations it can be concluded that no fitness cost in vegetative traits and reproductive ability was found in the R sub-population.

### Field Experiment

Similar to the greenhouse experiments, the fitness traits (biomass and tiller number) decreased significantly for both sub-populations with increasing densities of winter wheat. Also, no statistically significant differences were found between the R and S sub-populations in the measured fitness traits either in the absence or presence of winter wheat (**Table 3** and Supplementary Figure S4) confirming the results of the glasshouse experiments.

**Table 3:** Competitive responses of S and NTSR sub-populations selected within a black-grass population (ID914) to increasing density of winter wheat (neighbor plants) in field experiment.

	*ED_50_* (winter wheat plants m^-2^)^a^			*ED_90_* (winter wheat plants m^-2^)^b^			*D*^c^		
	*S*		*R*	*S*		*R*	*S*		*R*
Biomass	77 (27.6)	*ns*	148 (55.7)	697 (249)	*ns*	1334 (501)	31 (7.3)	*ns*	24 (5.1)
Tiller No.	120 (37.6)	*ns*	161 (50.0)	1080 (339)	*ns*	1452 (450)	101 (16.4)	*ns*	114 (17.2)

*^a^Effective density of wheat reducing response of target plants by 50%. ^b^Effective density of wheat reducing response of target plants by 90%. ^c^Mean response of target plants in the absence of winter wheat. *^ns^*non-significant (no significant differences between the R and S sub-populations). The parameters were obtained by fitting a nonlinear hyperbola model to data. Values in parentheses represent standard error of the estimated parameters.*

The potential seed production of plants was not evaluated in the field experiment as we did not want to contribute to the spread of herbicide resistance genes. However, according to the strong relationship usually found between vegetative and reproductive outputs of plants (Weiner, 2004; Weiner et al., 2009), the absence of significant differences in biomass and number of tillers suggest that the R and S sub-populations would also produce similar number of seeds, as it was also observed in the glasshouse experiments.

## Discussion

Many studies have investigated the fitness cost of herbicide resistance, however, in 75% of the published studies the genetic background of plants was not controlled possibly leading to flawed results (Vila-Aiub et al., 2009b), while it was revealed that greater control of genetic background increases the probability of identifying resistance costs (Bergelson and Purrington, 1996). Most of the studies that used appropriate protocols to control the genetic background have focused on fitness cost of TSR plants (Vila-Aiub et al., 2005b; Menchari et al., 2008; Ashigh and Tardif, 2009; Wang et al., 2010; Yu et al., 2010; Délye et al., 2013; Giacomini et al., 2014; Vila-Aiub et al., 2014). For instance, no vegetative and fecundity fitness cost were observed in ACCase-TSR black-grass phenotypes carrying the Leu-1781 and Asn-2041 ACCase mutations, while homozygous Gly-2078 ACCase black-grass showed a substantial fecundity and vegetative reduction (Menchari et al., 2008). Four populations of ALS-TSR black nightshade (*Solanum ptychanthum* Dunal) with mutation at position Ala-205 showed negative fecundity fitness, while they did not show any vegetative and seed germinability fitness penalty (Ashigh and Tardif, 2009; Ashigh and Tardif, 2011). High amplification of the 5-enolpyruvylshikimate-3-phosphate synthase (EPSPS) gene as a TSR mechanism endowing resistance to glyphosate in palmer amaranth and perennial ryegrass (*Lolium perenne* L.) was not associated with any significant reproductive fitness penalty (Giacomini et al., 2014; Vila-Aiub et al., 2014; Yanniccari et al., 2016). Surprisingly, seed production of an ACCase-TSR green foxtail phenotype carrying the Leu-1781 mutation was higher than the wild phenotype (Wang et al., 2010). Possible effects of TSR mechanism on plant fitness were investigated in all above mentioned studies showing that different resistance alleles and genes might have different finesses, i.e., negative, neutral or positive fitness.

Despite importance of NTSR mechanism, generally resistance costs of NTSR biotypes have rarely been studied in a correct way. So far, rigid ryegrass (*Lolium rigidum*) and loose silky bentgrass (*Apera spica-venti*) are the only NTSR weed species where fitness cost has been evaluated using appropriate plant material, where genetic background of plants were controlled via the Single Population and the Segregating Population approaches, respectively (Vila-Aiub et al., 2005a,b, 2009a; Pedersen et al., 2007; Babineau et al., 2017). This and our previous study (Keshtkar et al., 2017) are the first ones comparing S and NTSR sub-populations isolated from a resistant black-grass population using the Single Population Protocol providing random homogenous genetic background, as proposed by Vila-Aiub et al. (2011). In the present study, results from two greenhouse experiments and one field trial showed that NTSR mechanism conferring a high level of resistance to fenoxaprop-*P*-ethyl and a moderate level of resistance to pendimethalin and flupyrsulfuron was not associated with any vegetative and potential fecundity fitness cost. In contrast to our results, studies on an enhanced metabolic NTSR rigid ryegrass population resistant to ACCase inhibitors found fitness costs in both vegetative and reproductive outputs in competition with wheat (Vila-Aiub et al., 2005a, 2009a). In agreement with our results, no significant differences were reported in vegetative growth and seed production of S and glyphosate NTSR rigid ryegrass exposed to competition with wheat (Pedersen et al., 2007). In contrast to present study and all other studies evaluated fitness cost of NTSR weed species with appropriate methods of controlling genetic background (Vila-Aiub et al., 2005a,b, 2009a; Pedersen et al., 2007), recently Babineau et al. (2017) did not observe vegetative and fecundity fitness cost in an enhanced metabolic NTSR loose silky bentgrass population and surprisingly some fitness benefits (i.e., earlier germination and earlier flowering time) were observed. These results suggest that also for NTSR it is not possible to make any generalization and each case must be evaluated individually as it was suggested by Lehnhoff et al. (2013). It is worth to note that the exact mechanism endowing NTSR to black-grass and rigid ryegrass were not yet detected hitherto in our study and the study by Pedersen et al. (2007) and remained to be tested. However, we assumed that the enhanced metabolism might be the mechanism endowing NTSR in this population as it was reported in many black-grass populations and importantly the other NTSR mechanisms such as reduced uptake and translocation of herbicide were not detected in black-grass (Kemp et al., 1990; Hall et al., 1995; Menendez and De Prado, 1996; Hall et al., 1997; Letouzé and Gasquez, 2001). It was also suggested that increased herbicide metabolism is the main mechanism in 75% of French black-grass populations evolved resistance to ACCase inhibitors (Délye et al., 2007).

The results are significant because potential seed production, as a major component of plant fitness complex (Menchari et al., 2008; Vila-Aiub et al., 2009b) was evaluated over 2 years under competitive and non-competitive conditions and no differences were found between the S and NTSR sub-populations. Similar seed production potential does, however, not necessarily lead to equal fitness. Fitness of off-springs can be affected by seed germination pattern, seed dispersal, seed longevity, response to pathogens and diseases (Vila-Aiub et al., 2009b). Many pests and diseases attack black-grass that might influence seed production and viability (Moss, 1983). Also, Moss (1983) found a significant relationship between black-grass seed weight and seed viability as the seeds with higher weight had higher seed viability. Black-grass seed dormancy, germinability and rate were affected by cropping system, so that seeds originated from spring crop had lower dormancy and higher germinability (Colbach and Dürr, 2003). Seeds of black-grass produced under dry and warm conditions, also, had lower dormancy (Swain et al., 2006). According to these information, it is suggested that seed weight, seed viability, primary seed dormancy of R and S phenotypes must be measured under contrast conditions in future studies.

It was also suggested that many fitness traits should be considered to avoid misleading conclusions (Giacomini et al., 2014), especially under different conditions. Our previous study provided clear evidence for this, as seedling emergence of the NTSR plants was lower at sub-optimal conditions (low temperature and deep sowing depth) suggesting that the NTSR loci conferring herbicide resistance in the black-grass population were associated with negative fitness (Keshtkar et al., 2017). It should be noted that fitness penalties were only observed under stressful condition. Thus, we can only conclude that NTSR loci conferring resistance to black-grass were not associated with fitness penalty with regard to biomass production, number of tillers and potential seed production under either non-competitive or competitive conditions. Even it was not possible to measure effective see production, however, it is expected that the potential seed production estimated indirectly was a powerful index to measure fecundity fitness of plants. Also, it is said that black-grass seed production linked to the number of tillers (Maréchal et al., 2012). In addition, usually there is a strong relationship between vegetative and reproductive outputs of plants (Weiner, 2004; Weiner et al., 2009).

Environmental condition can affect fitness cost (Menchari et al., 2008), i.e., our results cannot be generalized to other stress conditions such as pathogen and pest infestations and maybe more importantly to abiotic stress conditions including drought, salinity, and cold. It has been speculated that NTSR biotypes, where resistance is attributed to an increased activity of detoxifying enzymes like cytochrome P-450s and glutathione transferases (GSTs), may possess a higher fitness than the wild type (Cummins et al., 2013; Taylor et al., 2013). For instance, wheat seedlings with induced GST had higher fitness (i.e., seedling length, dry weight and germination rate) than untreated plants under optimum as well as stressful conditions such as a soil contaminated with oil residues and heavy metals (Taylor et al., 2013). So, further studies are needed to confirm the absence of fitness cost under the stressful conditions.

Our study clearly highlighted the effect of crop density on black-grass suppression. Obviously, quantification of these effects can be applied in weed management strategies. On average increasing winter wheat density from 192 to 385 and 192 to 770 plant^-2^ reduced black-grass potential seed production around 50 and 70%, respectively. Other studies have also confirmed the effect of crop density on black-grass, for instance Lutman et al. (2013) reported a decrease in the number of black-grass head m^-2^ up to 37.5% when winter wheat crop density increased from 100 to 350 m^-2^, while the number of black-grass seedlings m^-2^ was not affected. It must be taken into account that yield and growth of crops can also be affected by the crop density. Optimal wheat density (i.e., the maximum number of plant m^-2^ without yield reduction) depends on many factors such as verity, climate, planting date, special planting pattern. For instance, in Australia the current recommended wheat density is 100–150 plant m^-2^ (Lemerle et al., 2005), while in a Mediterranean climate (Spain) the highest winter wheat yield was obtained at 400–500 seed m^-2^ and it was expected that higher densities might be suggestible (Lloveras et al., 2004). In Denmark, the recommended wheat density is around 250 and 400 plant m^-2^ for early and delayed sowing dates, respectively. Comparing the recommended crop densities with the ED_50_s presented in the **Table 3**, which is less than 200 plant m^-2^ for both biomass and tiller number, shows the importance of crop density as a herbicide resistance management tool. Another interesting result relates to the growth stage of winter wheat with plants at the 3–4 leaf stage being much more competitive than those at the 2-leaf stage. Effect of crop growth stage was, however, less pronounced than crop density. Overall, in the absence of competition black-grass could potentially produce 59,612 (±6,862) and 63,591 (±3,906) seeds per plant (when the planted winter wheat had 2- and 3–4 leaves, respectively), while the potential seed production of plants exposed to the highest density of winter wheat was 4,293 (±547) and 233 (±69) seeds per plant at 2-leaf and 3–4 leaf stage of winter wheat, respectively (the presented data are raw values, i.e., they were not calculated by the hyperbola model). It should be noted, that potential seed production in this study comprised both viable and non-viable seeds and the reduction in the percentage of viable seeds could be less or higher than the corresponding reduction in potential seed production. Our results suggest that agronomical practices leading to faster establishment of crop could be used to prevent a build-up of the black-grass seed bank. Also increasing the seeding density of winter wheat is an agronomic practice that can be used in an integrated weed management (IWM) strategy.

## Conclusion

In this study, no significant vegetative and reproductive fitness cost was found with the NTSR black-grass sub-population. Thus, the results of this study do not support the theory of resource-based allocation even under a stressful condition which is expected to magnify fitness cost (Vila-Aiub et al., 2009b). Hence it can be concluded that if effective herbicide resistant management strategies are not implemented, NTSR loci conferring resistance to a range of herbicides in black-grass will persist in the gene pool even after cessation of herbicide use. Consequently, herbicide as an effective tool for control of black-grass will gradually be lost in fields infested by NTSR black-grass. The results of this study revealed that NTSR black-grass sub-population can be expected to persist in the field within population and attention should, therefore, be devoted to IWM programs incorporating herbicide resistant management strategies. The results of this and a previous study (Keshtkar et al., 2017) have highlighted that all aspects of fitness traits should be measured in resistant plants.

## Author Contributions

EK, SM, and PK conceived and designed the experiments. EK conducted the experiments. EK analyzed the data. EK wrote the manuscript. EK, SM, and PK read and approved the manuscript.

## Conflict of Interest Statement

The authors declare that the research was conducted in the absence of any commercial or financial relationships that could be construed as a potential conflict of interest.

## References

[B1] Ashigh J. Tardif F. J. (2009). An amino acid substitution at position 205 of acetohydroxyacid synthase reduces fitness under optimal light in resistant populations of *Solanum ptychanthum*. *Weed Res.* 49 479–489. 10.1111/j.1365-3180.2009.00717.x

[B2] Ashigh J. Tardif F. J. (2011). Water and temperature stress impact fitness of acetohydroxyacid synthase–inhibiting herbicide-resistant populations of eastern black nightshade (*Solanum ptychanthum*). *Weed Sci.* 59 341–348. 10.1614/WS-D-10-00126.1

[B3] Babineau M. Mathiassen S. K. Kristensen M. Kudsk P. (2017). Fitness of ALS-inhibitors herbicide resistant population of loose silky bentgrass (*Apera spica-venti*). *Front. Plant Sci.* 8:1660. 10.3389/fpls.2017.01660 28993787 PMC5622297

[B4] Bergelson J. Purrington C. B. (1996). Surveying patterns in the cost of resistance in plants. *Am. Nat.* 148 536–558. 10.1086/285938

[B5] Brazier M. Cole D. J. Edwards R. (2002). O-Glucosyltransferase activities toward phenolic natural products and xenobiotics in wheat and herbicide-resistant and herbicide-susceptible black-grass (*Alopecurus myosuroides*). *Phytochemistry* 59 149–156. 10.1016/S0031-9422(01)00458-7 11809449

[B6] Carroll R. J. Ruppert D. (1988). *Transformation and Weighting in Regression.* New York, NY: Chapman and Hall.

[B7] Cavan G. Cussans J. Moss S. R. (2000). Modelling different cultivation and herbicide strategies for their effect on herbicide resistance in *Alopecurus myosuroides*. *Weed Res.* 40 561–568. 10.1046/j.1365-3180.2000.00211.x

[B8] Chauvel B. Guillemin J. Colbach N. Gasquez J. (2001). Evaluation of cropping systems for management of herbicide-resistant populations of blackgrass (*Alopecurus myosuroides* Huds.). *Crop Prot.* 20 127–137. 10.1016/S0261-2194(00)00065-X

[B9] Chauvel B. Munier-Jolain N. M. Grandgirard D. Gueritaine G. (2002). Effect of vernalization on the development and growth of *Alopecurus myosuroides*. *Weed Res.* 42 166–175. 10.1046/j.1365-3180.2002.00276.x

[B10] Colbach N. Dürr C. (2003). Effects of seed production and storage conditions on blackgrass (*Alopecurus myosuroides*) germination and shoot elongation. *Weed Sci.* 51 708–717. 10.1614/P2002-051

[B11] Cummins I. Cole D. J. Edwards R. (1999). A role for glutathione transferases functioning as glutathione peroxidases in resistance to multiple herbicides in black-grass. *Plant J.* 18 285–292. 10.1046/j.1365-313X.1999.00452.x 10377994

[B12] Cummins I. Wortley D. J. Sabbadin F. He Z. Coxon C. R. Straker H. E. (2013). Key role for a glutathione transferase in multiple-herbicide resistance in grass weeds. *Proc. Natl. Acad. Sci. U.S.A.* 110 5812–5817. 10.1073/pnas.1221179110 23530204 PMC3625300

[B13] Délye C. (2005). Weed resistance to acetyl coenzyme A carboxylase inhibitors: an update. *Weed Sci.* 53 728–746. 10.2307/4047044

[B14] Délye C. Menchari Y. Guillemin J. P. MatÉJicek A. Michel S. Camilleri C. (2007). Status of black grass (*Alopecurus myosuroides*) resistance to acetyl-coenzyme A carboxylase inhibitors in France. *Weed Res.* 47 95–105. 10.1111/j.1365-3180.2007.00544.x

[B15] Délye C. Menchari Y. Michel S. Cadet E. Le Corre V. (2013). A new insight into arable weed adaptive evolution: mutations endowing herbicide resistance also affect germination dynamics and seedling emergence. *Ann. Bot.* 111 681–691. 10.1093/aob/mct018 23393095 PMC3605953

[B16] Giacomini D. Westra P. Ward S. M. (2014). Impact of genetic background in fitness cost studies: an example from glyphosate-resistant palmer amaranth. *Weed Sci.* 62 29–37. 10.1614/WS-D-13-00066.1

[B17] Gronwald J. W. (1994). “Resistance to photosystem II inhibiting herbicides,” in *Herbicide Resistance in Plants. Biology and Biochemistry* eds Powles S. B. Holtum J. A. M. (New York, NY: CRC Press) 27–60.

[B18] Hall L. M. Moss S. R. Powles S. B. (1995). Mechanism of resistance to chlorotoluron in two biotypes of the grass weed *Alopecurus myosuroides*. *Pestic. Biochem. Physiol.* 53 180–192. 10.1006/pest.1995.1066

[B19] Hall L. M. Moss S. R. Powles S. B. (1997). Mechanisms of resistance to aryloxyphenoxypropionate herbicides in two resistant biotypes of *Alopecurus myosuroides* (blackgrass): herbicide metabolism as a cross-resistance mechanism. *Pestic.* *Biochem. Physiol.* 57 87–98. 10.1006/pest.1997.2259

[B20] Heap I. (2017). *The International Survey of Herbicide Resistant Weeds.* Available: www.weedscience.org [accessed June, 2016]

[B21] Holm L. Doll J. Holm E. Pancho J. V. Herberger J. P. (1997). *World Weeds: Natural Histories and Distribution.* New York, NY: John Wiley and Sons.

[B22] Holt J. S. (1990). “Fitness and ecological adaptability of herbicide-resistant biotypes,” in *Managing Resistance to Agrochemicals* eds Green M. B. LeBaron H. M. Moberg W. K. (Washington, DC: American Chemical Society) 419–429.

[B23] Hull R. Marshall R. Tatnell L. Moss S. (2008). Herbicide-resistance to mesosulfuron + iodosulfuron in *Alopecurus myosuroides* (black-grass). *Commun. Agric. Appl. Biol. Sci.* 73 903–912. 19226842

[B24] Kaiser Y. I. Gerhards R. (2015). Degradation and metabolism of fenoxaprop and mesosulfuron + iodosulfuron in multiple resistant blackgrass (*Alopecurus myosuroides*). *Gesunde Pflanzen* 67 109–117. 10.1007/s10343-015-0343-3

[B25] Kaundun S. S. (2014). Resistance to acetyl-CoA carboxylase-inhibiting herbicides. *Pest. Manag. Sci.* 70 1405–1417. 10.1002/ps.3790 24700409

[B26] Kemp M. S. Moss S. R. Thomas T. H. (1990). “Herbicide resistance in *Alopecurus myosuroides*,” in *Managing Resistance to Agrochemicals* eds Green M. B. LeBaron H. M. Moberg W. K. (Washington, DC: American Chemical Society) 376–393.

[B27] Keshtkar E. (2015). *Ecological Fitness, Molecular Basis and Selection of Resistant Black-Grass (Alopecurus myosuroides) Biotypes.* Ph.D. dissertation, Aarhus University Aarhus.

[B28] Keshtkar E. Mathiassen S. K. Beffa R. Kudsk P. (2017). Seed germination and seedling emergence of blackgrass (*Alopecurus myosuroides*) as affected by non–target-site herbicide resistance. *Weed Sci.* 65 732–742. 10.1017/wsc.2017.44

[B29] Keshtkar E. Mathiassen S. K. Moss S. R. Kudsk P. (2015). Resistance profile of herbicide-resistant *Alopecurus myosuroides* (black-grass) populations in Denmark. *Crop Prot.* 69 83–89. 10.1016/j.cropro.2014.12.016

[B30] Lehnhoff E. A. Keith B. K. Dyer W. E. Menalled F. D. (2013). Impact of biotic and abiotic stresses on the competitive ability of multiple herbicide resistant wild oat (*Avena fatua*). *PLOS ONE* 8:e64478. 10.1371/journal.pone.0064478 23696896 PMC3655980

[B31] Lemerle D. Cousens R. D. Gill G. S. Peltzer S. J. Moerkerk M. Murphy C. E. (2005). Reliability of higher seeding rates of wheat for increased competitiveness with weeds in low rainfall environments. *J. Agric. Sci.* 142 395–409. 10.1017/S002185960400454X

[B32] Letouzé A. Gasquez J. (2001). Inheritance of fenoxaprop-P-ethyl resistance in a blackgrass (*Alopecurus myosuroides* Huds.) population. *Theor. Appl. Genet.* 103 288–296. 10.1007/s001220100607

[B33] Lloveras J. Manent J. Viudas J. Lopez A. Santiveri P. (2004). Seeding rate influence on yield and yield components of irrigated winter wheat in a Mediterranean climate. *Agron. J.* 96 1258–1265. 10.2134/agronj2004.1258

[B34] Lutman P. J. W. Moss S. R. Cook S. Welham S. J. (2013). A review of the effects of crop agronomy on the management of *Alopecurus myosuroides*. *Weed Res.* 53 299–313. 10.1111/wre.12024

[B35] Maréchal P. Henriet F. Vancutsem F. Bodson B. (2012). Ecological review of black-grass (*Alopecurus myosuroides* Huds.) propagation abilities in relationship with herbicide resistance. *Biotechnol. Agron. Soc. Environ.* 16 103–113.

[B36] Melander B. (1995). Impact of drilling date on *Apera spica-venti* L. and Alopecurus myosuroides Huds, in winter cereals. *Weed Res.* 35 157–166. 10.1111/j.1365-3180.1995.tb02029.x

[B37] Menchari Y. Chauvel B. Darmency H. Délye C. (2008). Fitness costs associated with three mutant acetyl-coenzyme A carboxylase alleles endowing herbicide resistance in black-grass *Alopecurus myosuroides*. *J. Appl. Ecol.* 45 939–947. 10.1111/j.1365-2664.2008.01462.x

[B38] Menendez J. De Prado R. (1996). Diclofop-methyl cross-resistance in a chlorotoluron-resistant biotype of *Alopecurus myosuroides*. *Pestic. Biochem. Physiol.* 56 123–133. 10.1006/pest.1996.0066

[B39] Moss S. (2010). Integrated weed management (IWM): Will it reduce herbicide use? *Commun. Agric. Appl. Biol. Sci.* 75 9–17. 21542466

[B40] Moss S. R. (1983). The production and shedding of *Alopecurus myosuroides* huds seeds in winter cereals crops. *Weed Res.* 23 45–51. 10.1111/j.1365-3180.1983.tb00519.x

[B41] Moss S. R. (1999). *The Rothamsted Rapid Resistance Test for Detecting Herbicide-Resistance in Black-Grass, Wild-Oats and Italian Rye-Grass.* Available at: http://resources.rothamsted.ac.uk/black-grass-and-herbicide-resistance/rothamsted-rapid-resistance-test-detecting-herbicide-resistance

[B42] Moss S. R. Perryman S. A. M. Tatnell L. V. (2007). Managing Herbicide-resistant blackgrass (*Alopecurus myosuroides*): theory and practice. *Weed Technol.* 21 300–309. 10.1614/wt-06-087.1

[B43] Owen M. D. K. (2016). Diverse approaches to herbicide-resistant weed management. *Weed Sci.* 64 570–584. 10.1614/WS-D-15-00117.1

[B44] Park K. W. Mallory-Smith C. A. Ball D. A. Mueller-Warrant G. W. (2004). Ecological fitness of acetolactate synthase inhibitor–resistant and –susceptible downy brome (*Bromus tectorum*) biotypes. *Weed Sci.* 52 768–773. 10.1614/ws-04-081r 26258120

[B45] Pedersen B. P. Neve P. Andreasen C. Powles S. B. (2007). Ecological fitness of a glyphosate-resistant *Lolium rigidum* population: growth and seed production along a competition gradient. *Basic Appl. Ecol.* 8 258–268. 10.1016/j.baae.2006.01.002

[B46] Preston C. (2004). Herbicide resistance in weeds endowed by enhanced detoxification: complications for management. *Weed Sci.* 52 448–453. 10.1614/p2002-168b

[B47] Pye A. (2000). *The Regenerative Development of Alopecurus myosuroides Huds.* Available at: http://www.vaxteko.nu/html/sll/slu/ex_arb_ekologi_vaxtproduktion/EEV29/EEV29.HTM

[B48] R Core Team (2013). *R: A Language and Environment for Statistical Computing.* Vienna: R Foundation for Statistical Computing.

[B49] Ritz C. Baty F. Streibig J. C. Gerhard D. (2016). Dose-response analysis using R. *PLOS ONE* 10:e0146021. 10.1371/journal.pone.0146021 26717316 PMC4696819

[B50] Ritz C. Streibig J. C. (2005). Bioassay analysis using R. *J. Stat. Softw.* 12 1–22. 10.18637/jss.v012.i05

[B51] Swain A. J. Hughes Z. S. Cook S. K. Moss S. R. (2006). Quantifying the dormancy of *Alopecurus myosuroides* seeds moisture produced by plants exposed to different soil regimes temperature. *Weed Res.* 46 470–479. 10.1111/j.1365-3180.2006.00532.x

[B52] Taylor V. L. Cummins I. Brazier-Hicks M. Edwards R. (2013). Protective responses induced by herbicide safeners in wheat. *Environ. Exp. Bot.* 88 93–99. 10.1016/j.envexpbot.2011.12.030 23564986 PMC3608025

[B53] Tranel P. J. Wright T. R. Heap I. M. (2017). *Mutations in Herbicide-Resistant Weeds to ALS Inhibitors.* Available at: http://www.weedscience.com [accessed October 18 2017]

[B54] Vila-Aiub M. M. Goh S. S. Gaines T. A. Han H. Busi R. Yu Q. (2014). No fitness cost of glyphosate resistance endowed by massive EPSPS gene amplification in *Amaranthus palmeri*. *Planta* 239 793–801. 10.1007/s00425-013-2022-x 24385093

[B55] Vila-Aiub M. M. Neve P. Powles S. B. (2005a). Resistance cost of a cytochrome P450 herbicide metabolism mechanism but not an ACCase target site mutation in a multiple resistant *Lolium rigidum* population. *New Phytol.* 167 787–796. 10.1111/j.1469-8137.2005.01465.x 16101915

[B56] Vila-Aiub M. M. Neve P. Powles S. B. (2009a). Evidence for an ecological cost of enhanced herbicide metabolism in *Lolium rigidum*. *J. Ecol.* 97 772–780. 10.1111/j.1365-2745.2009.01511.x

[B57] Vila-Aiub M. M. Neve P. Powles S. B. (2009b). Fitness costs associated with evolved herbicide resistance alleles in plants. *New Phytol.* 184 751–767. 10.1111/j.1469-8137.2009.03055.x 19825013

[B58] Vila-Aiub M. M. Neve P. Roux F. (2011). A unified approach to the estimation and interpretation of resistance costs in plants. *Heredity* 107 386–394. 10.1038/hdy.2011.29 21540885 PMC3199924

[B59] Vila-Aiub M. M. Neve P. Steadman K. J. Powles S. B. (2005b). Ecological fitness of a multiple herbicide-resistant *Lolium rigidum* population: dynamics of seed germination and seedling emergence of resistant and susceptible phenotypes. *J. Appl. Ecol.* 42 288–298. 10.1111/j.1365-2664.2005.01017.x

[B60] Wang T. Picard J. C. Tian X. Darmency H. (2010). A herbicide-resistant ACCase 1781 *Setaria* mutant shows higher fitness than wild type. *Heredity* 105 394–400. 10.1038/hdy.2009.183 20087387

[B61] Weiner J. (2004). Allocation, plasticity and allometry in plants. *Perspect. Plant Ecol. Evol. Syst.* 6 207–215. 10.1078/1433-8319-00083

[B62] Weiner J. Campbell L. G. Pino J. Echarte L. (2009). The allometry of reproduction within plant populations. *J. Ecol.* 97 1220–1233. 10.1111/j.1365-2745.2009.01559.x

[B63] Yanniccari M. Vila-Aiub M. Istilart C. Acciaresi H. Castro A. M. (2016). Glyphosate resistance in perennial ryegrass (*Lolium perenne* L.) is associated with a fitness penalty. *Weed Sci.* 64 71–79. 10.1614/WS-D-15-00065.1

[B64] Yu Q. Han H. Vila-Aiub M. M. Powles S. B. (2010). AHAS herbicide resistance endowing mutations: effect on AHAS functionality and plant growth. *J. Exp. Bot.* 61 3925–3934. 10.1093/jxb/erq205 20627897 PMC2935867

