# Supplementary material for: No Vegetative and Fecundity Fitness Cost Associated with Acetyl-Coenzyme A Carboxylase Non-target-site Resistance in a Black-Grass (Alopecurus myosuroides Huds) Population
Source: Front Plant Sci. 2017 Nov 28;8:2011. doi: 10.3389/fpls.2017.02011 (PMC5712368; doi:10.3389/fpls.2017.02011)
Supplement: Supplementary file 1 [file Presentation_1.pdf]

*Supplementary Material*

# No vegetative and fecundity fitness cost associated with acetyl-coenzyme A carboxylase non-target-site resistance in a black-grass (*Alopecurus myosuroides* Huds) population

Eshagh Keshtkar[1, 2*], Solvejg K. Mathiassen[1], Per Kudsk[1]

[1]Department of Agroecology, Faculty of Science and Technology, Aarhus University, Slagelse, Denmark
[2]Department of Agronomy, Faculty of Agriculture, Tarbiat Modares University, Tehran, Iran

*** Correspondence***:*Eshagh Keshtkar, keshtkar@modares.ac.ir

**Supplementary Figures**

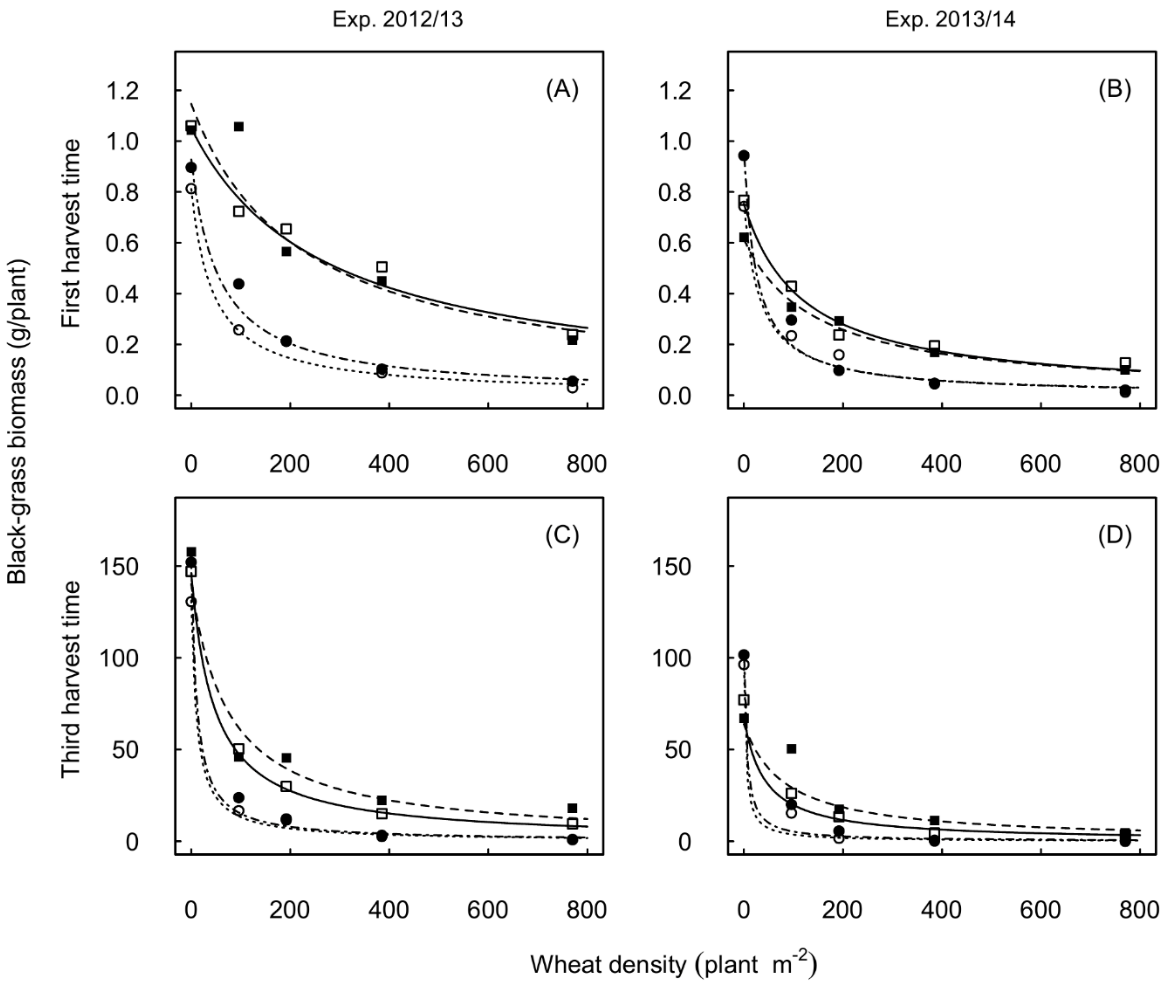


**Figure S1** Biomass production of herbicide-susceptible (S, open markers) and resistant (R, closed markers) black-grass sub-populations in response to increasing density and two different growth stages of winter wheat (2-leaf stage (GS-I); squares and 3-4 leaf stage (GS-II); circles) in glasshouse experiments 2012/13 (A and C) and 2013/14 (B and D). Data of first harvest time (A and B) and third harvest time (C and D) are presented. Estimated parameters are presented in Tables 1 (Experiment 2012/13) and 2 (Experiment 2013/14). The S and R sub-populations were selected within a NTSR population using a plant cloning technique.

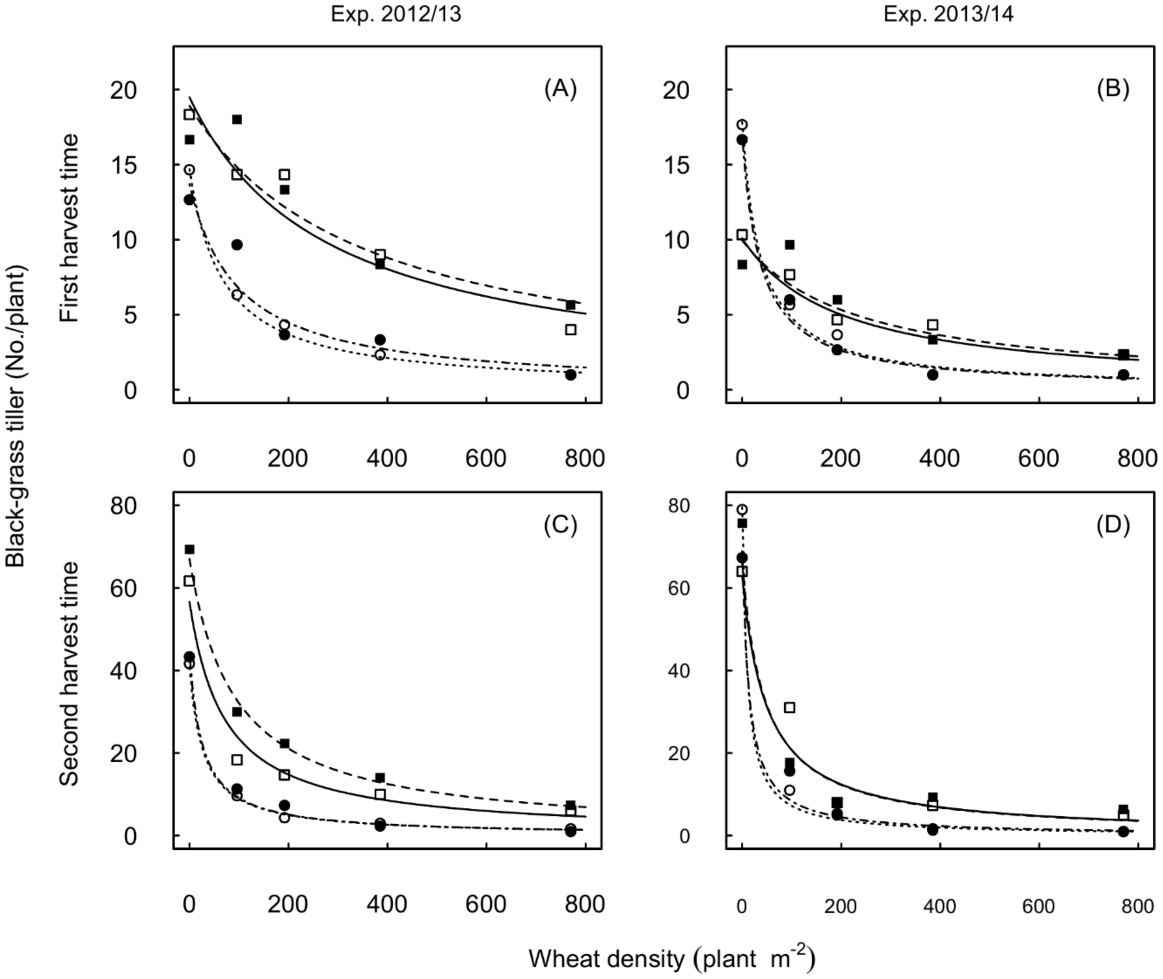


**Figure S2** Tiller production of herbicide-susceptible (S, open markers) and resistant (R, closed markers) black-grass sub-populations in response to increasing density and two different growth stages of winter wheat (2-leaf stage (GS-I); squares and 3-4 leaf stage (GS-II); circles) in glasshouse experiments 2012/13 (A and C) and 2013/14 (B and D). Data of first harvest time (A and B) and second harvest time (C and D) are presented. Estimated parameters are presented in Tables 1 (Experiment 2012/13) and 2 (Experiment 2013/14). The S and R sub-populations were selected within a NTSR population using a plant cloning technique.

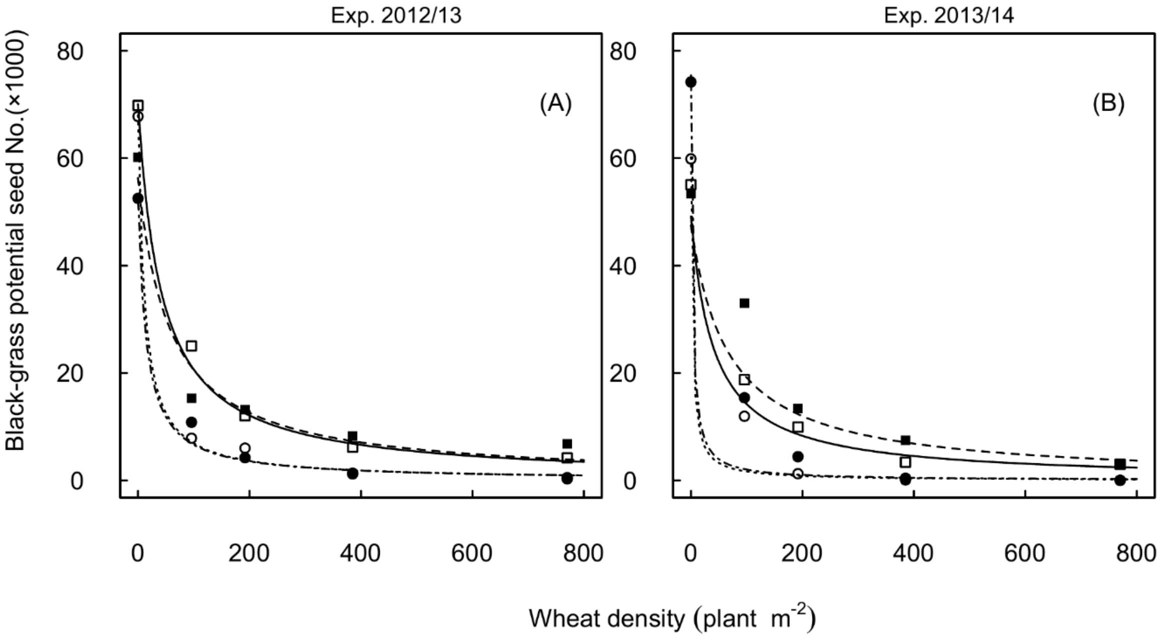


**Figure S3** Potential seed production of herbicide-susceptible (S, open markers) and resistant (R, closed markers) black-grass sub-populations in response to increasing density and two different growth stages of winter wheat (2-leaf stage (GS-I); squares and 3-4 leaf stage (GS-II); circles) in glasshouse experiments 2012/13 (A) and 2013/14 (B). Estimated parameters are presented in Tables 1 (Experiment 2012/13) and 2 (Experiment 2013/14). The S and R sub-populations were selected within a NTSR population using a plant cloning technique.

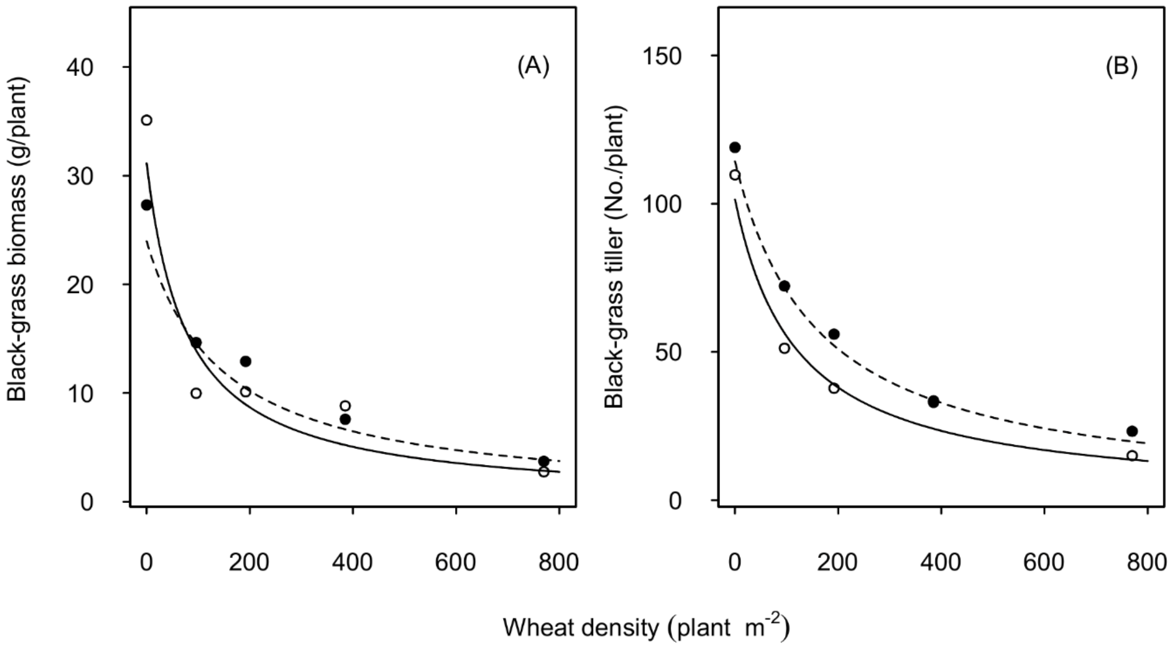


**Figure S4** Biomass (A) and tiller (B) production of herbicide-susceptible (S, open markers) and resistant (R, closed markers) black-grass sub-populations in response to increasing density of winter wheat in field experiment. Estimated parameters are presented in Table 3. The S and R sub-populations were selected within a NTSR population using a plant cloning technique.
